# Combining Multiple Imputation and Inverse-Probability Weighting

**DOI:** 10.1111/j.1541-0420.2011.01666.x

**Published:** 2012-03

**Authors:** Shaun R Seaman, Ian R White, Andrew J Copas, Leah Li

**Affiliations:** 1 MRC Biostatistics UnitCambridge, CB2 0SR, U.K.; 2 MRC Clinical Trials UnitLondon, NW1 2DA, U.K.; 3 Centre for Sexual Health and HIV Research, University College LondonLondon, WC1E 6JB, U.K.; 4 MRC Centre of Epidemiology for Child Health, UCL Institute of Child HealthLondon, WC1N 1EH, U.K.

**Keywords:** Marginal model, Missing at random, Survey weighting, 1958 British Birth Cohort

## Abstract

**Summary:**

Two approaches commonly used to deal with missing data are multiple imputation (MI) and inverse-probability weighting (IPW). IPW is also used to adjust for unequal sampling fractions. MI is generally more efficient than IPW but more complex. Whereas IPW requires only a model for the probability that an individual has complete data (a univariate outcome), MI needs a model for the joint distribution of the missing data (a multivariate outcome) given the observed data. Inadequacies in either model may lead to important bias if large amounts of data are missing. A third approach combines MI and IPW to give a doubly robust estimator. A fourth approach (IPW/MI) combines MI and IPW but, unlike doubly robust methods, imputes only isolated missing values and uses weights to account for remaining larger blocks of unimputed missing data, such as would arise, e.g., in a cohort study subject to sample attrition, and/or unequal sampling fractions. In this article, we examine the performance, in terms of bias and efficiency, of IPW/MI relative to MI and IPW alone and investigate whether the Rubin’s rules variance estimator is valid for IPW/MI. We prove that the Rubin’s rules variance estimator is valid for IPW/MI for linear regression with an imputed outcome, we present simulations supporting the use of this variance estimator in more general settings, and we demonstrate that IPW/MI can have advantages over alternatives. IPW/MI is applied to data from the National Child Development Study.

## 1. Introduction

Datasets collected for medical or social research contain missing values. One approach for dealing with this problem is simply to exclude individuals with missing data. This “complete-case” analysis is valid when data are missing completely at random but not necessarily when missing at random (MAR) (Little and Rubin, 2002). It can also be inefficient. Two alternatives are inverse-probability weighting (IPW) (Höfler et al., 2005) and multiple imputation (MI) (Little and Rubin, 2002). In IPW, again only complete cases are included in the analysis (excepting analysis of repeated measures, which we do not treat here), but weights are used to rebalance the set of complete cases so that it is representative of the whole sample. Inverse-probability weights can also be used to adjust for different sampling fractions in a survey. They are then known as sampling weights and rebalance the sample to make it representative of the population.

In MI, missing data are replaced by data drawn from an imputation model. This is done *M* times, generating *M* complete datasets. Each is analyzed and an estimate of the model parameters, 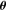, calculated. Let 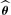 denote the complete-data estimator of 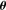, and 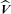 its estimated variance. Let 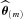 and 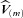 be their values for the *m*th imputed dataset (*m*= 1, … , *M*). Rubin (1987) proposed 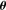 be estimated by 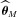 and 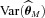 by 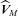, where 
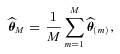
(1)

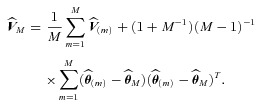
(2)

IPW and MI yield consistent estimators of 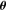 when the data are MAR and the imputation and weighting models, respectively, are correctly specified. The variance of the IPW estimator is consistently estimated provided the weighting is taken into account, e.g., using a sandwich estimator (Robins, Rotnitzky, and Zhao, 1994). For MI, when 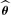 is the maximum likelihood estimator (MLE), 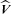 is the inverse Fisher information, and missing data are sampled from their Bayesian posterior predictive distribution, 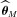 is asymptotically normally distributed with variance 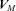, and 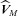 is an asymptotically unbiased estimator of 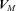 and is consistent when *M*=∞ (Rubin, 1987; Wang and Robins, 1998; Nielsen, 2003).

MI is often preferred to IPW, as it is usually more efficient. If the imputation model is correctly specified, MI should work well. However, if many data are being imputed, any inadequacies in the imputation model may lead to considerable bias. If few variables are missing on an individual, it may be considered desirable to impute them, rather than exclude the individual. On the other hand, if many variables are missing on the same individual, the imputation model must describe the joint distribution of all these variables, and if many individuals have many missing variables, the analyst may be nervous about relying on this complex and possibly misspecified imputation model. This situation could arise, for example, in a longitudinal study when whole blocks of data are missing on some of the individuals due to missed visits, or in a survey when some individuals have declined to answer whole sets of related questions. In such situations, the analyst may feel more confident using IPW.

Another possibility is to combine MI and IPW. A rule is specified for when to include an individual in the analysis: e.g., if they attended a follow-up visit, or if more than a certain percentage of their data is observed. Missing values in included individuals are multiply imputed and each resulting dataset (which we call a “quasi-complete dataset” because the data are complete for the included, but not excluded, individuals) is analyzed using IPW to account for the exclusion of individuals not satisfying the inclusion rule and for different sampling fractions (if any). The “quasi-complete-data” estimator 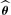 is then the IPW estimator using the data on included individuals in a single quasi-complete dataset and 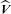 is the corresponding sandwich variance estimator. We call this method “IPW/MI.” By imputing in individuals with few missing values but excluding individuals with more missing data, IPW/MI could inherit some of the efficiency advantage of MI while avoiding bias resulting from incorrectly imputing larger blocks of data. IPW/MI is also needed when sampling weights are used together with MI, even if all individuals are included in the analysis.

Several authors have used IPW/MI. Caldwell et al. (2008) and Stansfeld et al. (2008a,2008b) analyzed data from the National Childhood Development Study (NCDS). They regressed outcomes measured at age 45 on predictors measured at the same or earlier visits. Attrition of the cohort over time meant that 41% missed the age 45 visit. Weights were used to adjust for attrition, while missing values in those who attended the visit were multiply imputed. Priebe et al. (2004) multiply imputed missing data in a logistic regression with sampling weights.

It is not obvious that Rubin’s rules will give valid variance estimators for IPW/MI. IPW estimators are inefficient. Robins and Wang (2000) and Nielsen (2003) show for MI that when 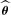 is inefficient, 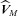 can be asymptotically biased, even if 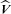 is a consistent estimator of the complete-data variance and imputation is from the correct posterior predictive distribution. The purpose of the present article is twofold: to examine asymptotic bias in 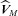 when 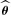 is an IPW estimator and to show when IPW/MI is useful.

In Section 2, we define IPW/MI and show it gives consistent estimation of 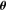. In Section 3, we show 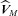 is asymptotically unbiased for IPW/MI with linear regression and imputed outcomes. Section 4 describes a simulation study verifying this and demonstrating IPW/MI can have advantages over MI or IPW alone. Section 5 is a simulation with imputed covariate, suggesting 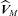 is approximately unbiased in this case. Section 6 is an application to NCDS.

## 2. IPW/MI and Consistency of 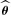


In this section, we describe IPW/MI for the situation where there are no sampling weights. The inclusion of sampling weights is covered in the Web Appendix available online.

An independent random sample of size *N* is drawn from the population. Let 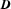 denote, for an individual, the vector of the set of variables included in the analysis model as well as possibly other variables that will be used to impute missing values in that set of variables. Let *R* denote the missingness pattern in 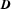 (i.e., which elements of 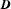 are missing), and write 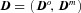, where 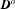 and 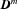 denote the observed and missing parts of 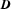, respectively. Subscript *i* denotes individual *i* in the sample; e.g., 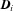 denotes 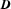 for individual *i*.

The IPW/MI method is as follows. Let 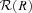 be a binary function of *R* chosen by the analyst. 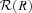 is the rule determining whether an individual is included in the analysis. An example of 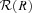 is 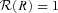 if fewer than a certain percentage of variables in the analysis model are missing and 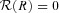 otherwise. Let 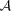 denote the set of indices of individuals with 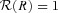. As formalized below, we estimate 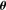 by fitting the analysis model only to individuals 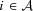, using inverse-probability weights to account for the selection by 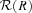. Missing values in individuals 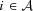 are multiply imputed.

To impute 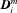 in individuals 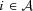, we assume a model 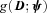 for the conditional distribution of 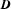 given 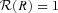 with parameters 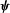. We say this model is correctly specified if 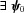 such that 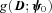 is the true distribution of 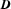 given 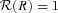. 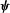 is estimated by 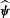, its MLE using only the data on individuals 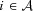. Imputation may be proper or improper. Let 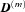 denote the *m*th imputed value of 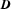 (*m*= 1, … , *M*). Note that if some elements of 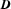 are observed in all individuals with 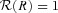, the imputation model can be a model for the distribution of the remaining elements of 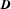 given these elements and 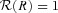.

Let 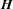 be a vector of fully observed variables that predict whether 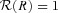. Assume a model 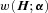 for 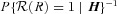, where 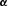 are parameters. We say this model is correctly specified if 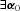 such that 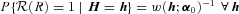. Let 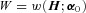. Assume ∃δ > 0 such that *P*(*W*^−1^ > δ) = 1. Typically, 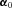, the true value of 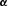, will be unknown. Let 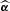 equal 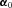 if 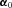 is known and denote the MLE of 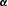 otherwise.

Let 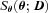 denote an individual’s contribution to the (unweighted) complete-data estimating equations of the analysis model. Let 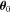 denote the solution of 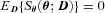. Therefore, 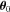 is the “true” value of 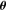: it is the value to which the solution to estimating equations 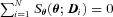 would converge as *N*→∞. Based just on data from individuals 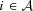, let 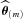 be the solution to (weighted) estimating equations 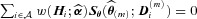 and let 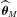 be given by equation (1). Theorem 1 and its corollary state that under specified conditions 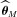 is a consistent estimator of 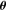. Proofs are given in the Web Appendix.

**theorem 1** *Assume (i) model*
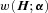
*is correctly specified, (ii)*
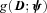
*is correctly specified, (iii)*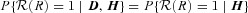, *(iv)*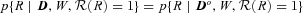, *and (v)*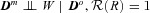. *Then, when*
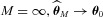
*as N*→∞.

Condition (iii) states that the probability an individual is used in the fitting of the imputation and analysis models does not depend on his values of the variables (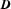) used in those models given the covariates (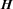) in the weighting model. Condition (iv) states that among individuals to whom the imputation model is fitted, 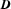 is MAR given the true weight *W*. Condition (v) adds to this that among these individuals the missing variables in the imputation model must be conditionally independent of *W* given the observed variables. Note condition (v) can be satisfied by including *W* or 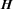 in 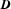. The necessity for condition (v) can be understood by considering how imputation will work if it is not satisfied. Set 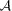 is enriched for individuals with small values of *W* (and contains fewer with large values) compared to the entire sample. If (v) is false, the distribution of 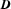 given *W* depends on *W*, and when the imputation model is fitted to set 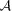, the resulting estimate of the marginal distribution of 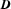 will be biased toward the conditional distribution of 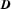 given small values of *W*. Missing data in all individuals in 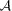 are then imputed using the same model, a model that has been estimated giving too much weight to individuals with small *W*. Including *W* (or 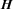) in the imputation model avoids this problem: individuals with different *W* are imputed differently.

The following corollary shows that an alternative to including the true weights (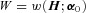) or the covariates that predict the weights (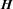) in the imputation model is to include the estimated weights (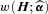). The latter may be appealing because true weights are typically unknown and the dimension of 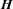 may be large.

**corollary 1** *Suppose the imputation model includes, in addition to*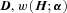. *Assume conditions (i), (iii), and (iv) of Theorem 1 are satisfied, the imputation model*
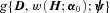
*is correctly specified,*
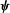
*is estimated by its MLE*
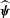
*at*
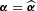
*using only individuals*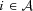, *and*
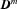
*is imputed using*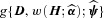. *Then, when*
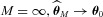
*as N*→∞.

## 3. Linear Regression with Imputed Outcome

Consider the special case of linear regression with an imputed outcome. As in Section 2, we assume that there are no sampling weights; the generalization to sampling weights is given in the Web Appendix. Write 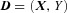 and let 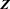 be 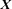 or a subvector of 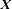. Below, *Y* and 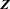 will be the response and covariates, respectively, in the analysis model. Let 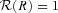 if 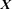 is complete; 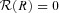 otherwise. Let *R_Y_*= 1 if 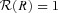 and *Y* is observed; *R_Y_*= 0 otherwise. We assume weights *W* are known and ∃δ > 0 such that *P*(*W*^−1^ > δ) = 1.

We estimate 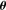 in the analysis model 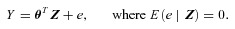
(3) by linear regression of *Y* on 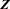. Therefore, 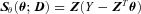. The true value of 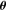 is the solution of 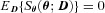, which is 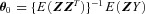. We say the analysis model is correctly specified if equation (3) holds 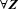 when 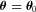; otherwise it is misspecified.

The quasi-complete-data estimator, 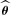, is the solution to weighted estimating equations 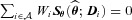, which is the weighted least squares estimator 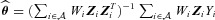. The quasi-complete-data variance estimator 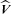 is the sandwich estimator 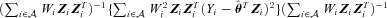. Missing *Y* values in individuals 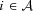 are multiply imputed using 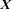 as predictors, 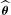 and 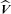 are calculated for each imputed dataset, and 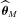 and 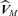 are calculated from equations (1) and (2).

**theorem 2** *Let missing Y be imputed from their posterior predictive distributions using the regression imputation procedure of* *Schenker and Welsh (1988)* *(p. 1560) with imputation model*

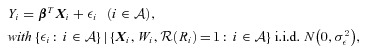
(4)
*and improper prior density for*
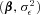
*proportional to*σ^−2^_ε_. *Assume this model is correctly specified, i.e., there exists a*
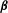
*for which* *equation (4)* *holds, and that*

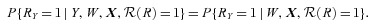
(5)

Then (i) 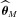 is a consistent estimator of 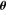; (ii) if 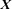 includes 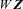 (i.e., 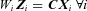 for some matrix of constants 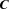), 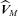 is an asymptotically (*N*→∞) unbiased estimator of 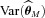; and iii) if 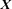 includes 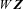 and 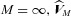 is a consistent estimator of 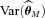.

Including 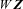 in 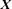 means including the pairwise interactions between the weight and all the variables in 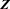, as well as (if the analysis model includes an intercept term) the weights themselves. Proofs of parts (i) and (ii) come from extending the proof of Kim et al. (2006), which shows (ii) is true in the special case where 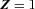; that of part (iii) comes from applying Theorem 2 of Robins and Wang (2000). Details are in the Web Appendix.

The reason 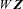 needs to be in 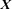 is to avoid the imputer assuming more than the analyst. Consider the simple case where 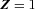 (so θ is the population mean) and there are two values of *W*: *a* and *b*. The complete-data estimator of θ corresponds to stratifying the sample by *W*, calculating the mean in each of the two strata and then calculating a weighted average of these two means. Thus, the analysis model does not assume the population mean is the same in the two strata. If the imputation model does not include *W*, it assumes the population mean is the same in the two strata, with the result that the imputer is assuming more than the analyst, which is known to lead to overestimation of the variance of 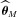 when the extra assumption made by the imputer is correct (Meng, 1994). If the true value of the coefficients of 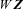 is zero, because the imputation model is correctly specified without the 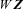 terms, it is probably better not to include these terms and instead accept some overestimation of 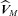: imputation will be more efficient if they are set to zero rather than estimated.

Note that, because 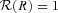 only if 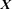 is complete, individuals with incomplete 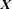 are excluded, even if their *Y* and 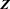 are complete. For this reason, it would not be appealing to use this method if the sample contained more than a few such individuals.

An alternative to IPW/MI is what we call “IPW/CC.” Here *Y* is regressed on 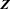 only in complete cases (those with *R_Y_*= 1), again using weights *W*. This estimator is unbiased if 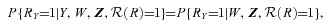
(6) and the analysis model is correctly specified. If weights *W* are all equal, and 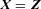 and the imputation and analysis models are the same, there is no benefit to IPW/MI over IPW/CC: it is more efficient to exclude individuals with missing *Y* (unless *M*=∞, in which case exclusion and imputation are equivalent) (White and Carlin, 2010). However, there are two reasons for preferring IPW/MI to IPW/CC. These apply whether or not weights are equal. First, if (6) does not hold or if the analysis model is misspecified, the complete-case estimator may be inconsistent, whereas, as Theorem 2 states, IPW/MI gives consistent estimators if equation (5) holds (and assuming the imputation model is correctly specified). Equation (5) may be satisfied even if (6) is not, as (5) allows the probability that *Y* is observed to depend on a larger set of variables 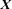. Second, even if (6) holds and the analysis model is correctly specified, it may be more efficient to use all the available information (i.e., 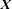) to impute *Y*.

## 4. Simulation Study: Imputed Outcome

In this section, we explore IPW/MI for linear regression with imputed outcome. As in Section 3, the analysis model is fitted only to individuals with complete 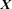 and missing *Y* in these individuals are imputed.

Analysis of the sample must deal with two stages of missingness: stage 1 is the missingness in 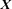; stage 2, missingness in *Y*. At stage 1, one could either exclude individuals with incomplete 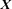 (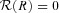) or impute missing 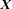. Similarly, each individual with missing *Y* not already excluded at stage 1 (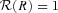) could either be excluded at stage 2 or have *Y* imputed. At each stage, if exclusion is used, one can either adjust for the exclusion using IPW or not adjust. Thus, there are three possibilities at each stage, giving 3 × 3 = 9 possible strategies in total. Denote a strategy by ST1/ST2, where ST1 and ST2 are each CC (exclude and do not weight), IPW (exclude and weight) or MI (impute). In IPW/MI, the focus of this article, individuals with missing 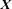 (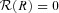) are excluded and weights used to adjust for this; individuals with complete 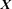 but missing *Y* (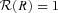) have *Y* imputed. CC/CC uses only individuals with complete 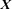 and *Y* and there is no weighting. IPW/IPW uses the same individuals, but weights them by the inverse of their probability of being a complete case. In MI/MI all missing values are imputed. We also consider CC/IPW, CC/MI, and IPW/CC, but not MI/CC or MI/IPW, which combine the disadvantage of having to specify an imputation model for 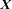 with that of losing out on the potential efficiency gains of imputing *Y*.

The purpose of the following simulation is three-fold: to verify 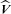 is approximately unbiased for IPW/MI; to show IPW/MI can be more efficient than IPW/IPW; and to show MI/MI can yield biased parameter estimators when the stage 1 (for 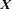) or stage 2 (for *Y* given 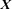) imputation model is misspecified, and that IPW/MI remains approximately unbiased or at least less biased than MI/MI in these situations. The data-generating mechanism has been chosen to illustrate these points. It will now be described and then its features elucidated.

Data 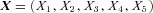 and *Y* were generated for *N*= 1000 individuals. For each individual, *X*_1_ was one with probability 0.5 and zero otherwise, *X*_2_, *X*_3_, and *X*_4_ were independent and identically distributed *N*(0, 1) and, finally, *X*_5_ was sampled from *N*(*X*_2_×*X*_3_, 1). Response *Y* was generated from 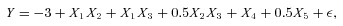
(7) where ε∼*N*(0, 1). *X*_1_ was observed for all *N* individuals. With probability 0.8 − 0.6*X*_1_, (*X*_2_, *X*_3_, *X*_4_, *X*_5_) was observed; otherwise it was missing. If (*X*_2_, *X*_3_, *X*_4_, *X*_5_) was observed, *Y* was observed with probability {1 + exp (−1.5 + 0.6*X*_2_*X*_4_)}^−1^; otherwise *Y* was missing.

The analysis model was *Y* = θ_0_ + θ_2_*X*_2_ + θ_3_*X*_3_ + θ_23_*X*_2_*X*_3_ + *e*, where *E*(*e* ∣*X*_2_, *X*_3_) = 0. Therefore, 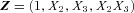. By integrating (7) with respect to *X*_1_, *X*_4_, and *X*_5_, it can be shown that this analysis model is correctly specified and the true 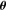 is (θ_0_, θ_2_, θ_3_, θ_23_) = (− 3, 0.5, 0.5, 1).

This data-generating mechanism was chosen for three reasons. First, the *X*_1_*X*_2_ and *X*_1_*X*_3_ interactions in (7) mean the relation between *Y* and (*X*_2_, *X*_3_) is different in the two strata defined by *X*_1_. Also, the probability that (*X*_2_, *X*_3_) is observed differs: in one stratum it is 0.2; in the other, 0.8. Thus, the relation between *Y* and (*X*_2_, *X*_3_) is different in individuals with complete 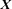 and incomplete 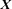. Failure to adjust for the missingness at stage 1, by weighting or imputation, will therefore lead to bias in θ_2_ and θ_3_. Therefore, CC/IPW, CC/MI, and CC/CC will be biased. Second, for individuals with observed (*X*_2_, *X*_3_, *X*_4_, *X*_5_) the probability *Y* is observed depends on *X*_4_, which is not in the analysis model but is associated with *Y*. This causes the relation between *Y* and 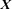 described by the analysis model to be different in the set of complete cases from in the set with complete 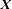 but missing *Y*. In particular, because the probability of *Y* being missing depends on *X*_2_*X*_4_, the relation between *Y* and *X*_2_ will be different in the two sets. Failure to adjust for the missingness at stage 2 will therefore lead to bias (specifically in θ_2_). Therefore, IPW/CC, MI/CC, and CC/CC will be biased. Third, *X*_5_ is included in the data-generating mechanism for *Y* to show that using MI at stage 1 can cause bias if the imputation model for 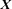 is misspecified (see results for MI*/MI below).

A total of 1000 datasets were generated and the seven methods applied to each. For each of θ_0_, θ_2_, θ_3_, and θ_23_ and each method, the mean of the 1000 parameter estimates and of the 1000 estimated variances was calculated. The empirical SE was calculated as the standard deviation of the parameter estimates. Where a method involved imputation, 10 imputations were performed.

For MI/MI, the (correctly specified) imputation model at stage 1 was (*X*_2_, *X*_3_, *X*_4_) ∼*N*{(γ_2_, γ_3_, γ_4_), Σ_1_} and *X*_5_∣*X*_2_, *X*_3_∼*N*(γ_5_+γ_6_*X*_2_+γ_7_*X*_3_+γ_8_*X*_2_*X*_3_ , Σ_2_). Noninformative normal and inverse-Wishart priors were used, yielding normal and inverse-Wishart posteriors (Gelman et al., 2004, p. 88). For CC/MI, IPW/MI, and MI/MI, the (correctly specified) imputation model used at stage 2 was *Y*=β_0_+β_1_*X*_1_+β_2_*X*_2_+β_3_*X*_3_+β_4_*X*_4_+β_5_*X*_5_+β_12_*X*_1_*X*_2_+β_13_*X*_1_*X*_3_+β_23_*X*_2_*X*_3_+β_123_*X*_1_*X*_2_*X*_3_+ε.

For IPW/CC, IPW/IPW, and IPW/MI, weights were estimated by fitting the (correctly specified) missingness model for stage 1: *P*(*X*_2_, *X*_3_, *X*_4_ and *X*_5_ observed) =δ_0_+δ_1_*X*_1_. Note that, because *X*_1_ is binary, *W*= (δ_0_+δ_1_*X*_1_)^−1^=δ^−1^_0_−*X*_1_δ_1_{δ_0_(δ_0_+δ_1_)}^−1^ is a linear function of *X*_1_. Hence, as the stage 2 imputation model includes 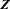 and 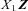, it implicitly includes 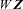. For CC/IPW and IPW/IPW, weights were estimated using the (correctly specified) model for stage 2: 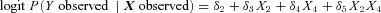. For IPW/IPW, the probability of being a complete case is the product of these two probabilities.

Table 1 shows mean parameter estimates, empirical SEs, and square roots of the mean estimated variances. It can be seen that IPW/MI yields approximately unbiased estimators of parameters and SEs, as expected from Theorem 2. As explained above, CC/IPW, CC/MI, CC/CC, and IPW/CC are biased for one or more parameters. IPW/IPW and MI/MI are both approximately unbiased. The former is less efficient than IPW/MI because the imputation model at stage 2 uses auxiliary information, i.e. covariates (notably *X*_4_ and *X*_5_) not included in the analysis model. The most efficient unbiased method is MI/MI, confirming that imputation is the best method when the imputation models are correct.

**Table 1:** *Mean parameter estimate (“mean”), square root of mean estimated variance (“aSE”), and empirical SE (“eSE”) for four parameters and 10 analysis methods. The true value of*
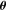
*is* (θ_0_, θ_2_, θ_3_, θ_23_) = (−3, 0.5, 0.5, 1).

	θ_0_			θ_2_			θ_3_			θ_23_		
				
Method	Mean	aSE	eSE	Mean	aSE	eSE	Mean	aSE	eSE	Mean	aSE	eSE
True	−3.000			.500			.500			1.000		
CC/CC	−2.995	.080	.079	.090	.081	.087	.200	.080	.086	1.005	.082	.091
CC/IPW	−2.993	.082	.079	.199	.092	.091	.200	.086	.089	1.004	.094	.100
CC/MI	−2.994	.075	.076	.202	.081	.081	.201	.079	.083	1.004	.084	.086
IPW/CC	−2.993	.102	.101	.382	.110	.112	.495	.109	.114	1.008	.114	.119
IPW/IPW	−2.990	.106	.104	.489	.120	.124	.494	.112	.117	1.006	.121	.132
IPW/MI	−2.992	.097	.096	.498	.105	.105	.497	.104	.107	1.006	.110	.113
MI/MI	−3.000	.089	.081	.503	.092	.087	.497	.090	.088	1.006	.092	.082
MI^*^/MI	−2.998	.092	.085	.498	.095	.093	.496	.094	.094	.749	.100	.083
MI/MI^*^	−2.999	.108	.101	.100	.088	.054	.099	.088	.051	.391	.091	.055
IPW/MI^*^	−2.998	.107	.100	.492	.119	.122	.495	.117	.115	.776	.131	.127

However, when the imputation model at stage 1 or stage 2 is misspecified, MI/MI may be biased. First, suppose that the imputation model at stage 1 is misspecified as (*X*_2_, *X*_3_, *X*_4_, *X*_5_)^*T*^∼*N*{(γ_2_, γ_3_, γ_4_, γ_5_)^*T*^ , Σ}. As *X*_2_, *X*_3_, *X*_4_ and *X*_5_ are uncorrelated (though not independent), Σ will be estimated as an approximately diagonal matrix. Therefore, for individuals with incomplete 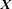 the imputed values of *X*_5_ will be approximately independent of *X*_2_ and *X*_3_; the relation between *X*_5_ and the interaction of *X*_2_ and *X*_3_ (*E*(*X*_5_) =*X*_2_*X*_3_) is not present in the imputed data. The missing *Y* values of these individuals will then be imputed in such a way that the interaction between *X*_2_ and *X*_3_ is only 0.5, half what it should be. As half the individuals have incomplete 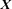, fitting the analysis model to the whole sample results in an estimate of θ_23_ of about 0.75. This is seen in Table 1 in the row MI*/MI.

Second, suppose the imputation model at stage 1 is correct but that at stage 2 is misspecified by leaving out the β_23_*X*_2_*X*_3_, β_123_*X*_1_*X*_2_*X*_3_, and β_5_*X*_5_ terms. Missing *Y* values will now be imputed in such a way that there is no interaction between *X*_2_ and *X*_3_. As approximately 60% of *Y* values are missing, θ_23_ will be underestimated by about 60%. This result is shown in Table 1 in the row MI/MI*. The row IPW/MI* shows the result of IPW/MI with the same misspecified imputation model at stage 2. This method is considerably less biased than MI/MI*, because fewer *Y* values are being imputed. Therefore, the IPW element of IPW/MI provides some protection against misspecification of the imputation model.

## 5. Simulation Study: Imputed Covariate

In this section, we investigate the bias of 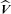 for IPW/MI in the case of linear regression with an imputed covariate. In the simulation study below, we find that the bias is small. This study also demonstrates again that IPW/MI can be more efficient than IPW/IPW, and that MI/MI can yield biased estimators when the imputation model for stage 1 is misspecified. Only brief details are presented here; full details can be found in the Web Appendix.

The (correctly specified) analysis model was *Y*=θ_0_+θ_2_*X*_2_+θ_3_*X*_3_+θ_4_*X*_4_+θ_23_*X*_2_*X*_3_+*e*, where *E*(*e* ∣*X*_2_, *X*_3_, *X*_4_) = 0. Variables *X*_1_ and *Y* were always observed; *X*_2_ and *X*_3_ were both observed or both missing. The probability they were observed depended on *Y* and *X*_1_. If (*X*_2_, *X*_3_) was missing, so was *X*_4_; otherwise the probability *X*_4_ was observed depended on *Y*. The two stages of missingness are that stage 1 is missingness in (*X*_2_, *X*_3_) and stage 2 is missingness in *X*_4_.

For MI/MI, the imputation model used at stage 1 (to impute *X*_2_ and *X*_3_) falsely assumed that (*Y*, *X*_2_, *X*_3_) was trivariate normal. Although misspecified, this imputation model might easily be used in practice. As the stage 1 imputation model is misspecified, we call this method MI*/MI. For IPW/MI and MI*/MI, the imputation model used at stage 2 (to impute *X*_4_) was correctly specified in terms of *X*_1_, *X*_2_, *X*_3_, *Y*, and certain interactions. The covariates (*X*_1_ and *Y*) that determine the weights are included in this model. IPW/MI* and MI*/MI* used a stage 2 imputation model that was misspecified because interaction terms were omitted.

Table 2 shows the results. IPW/IPW and IPW/MI are approximately unbiased, and SE estimators for IPW/MI are approximately unbiased. SEs for IPW/MI are smaller than for IPW/IPW: it is more efficient to impute missing *X*_4_ for individuals with otherwise complete data than to exclude them.

**Table 2:** *Mean parameter estimate (mean), square root of mean estimated variance (aSE), and empirical SE (eSE) for five parameters and 10 analysis methods. Results for*θ_2_*are omitted because, apart from Monte Carlo error, they are the same as for*θ_3_. *The true value of*
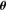
*is* (θ_0_, θ_2_, θ_3_, θ_4_, θ_23_) = (0, 0.5, 0.5, 0.5, 1).

	θ_0_			θ_3_			θ_4_			θ_23_		
				
Method	Mean	aSE	eSE	Mean	aSE	eSE	Mean	aSE	eSE	Mean	aSE	eSE
True	.000			.500			.500			1.000		
CC/CC	.238	.060	.056	.196	.061	.065	.183	.060	.064	.992	.064	.077
IPW/IPW	.020	.095	.102	.485	.103	.113	.479	.108	.124	.990	.108	.119
IPW/MI	.002	.075	.075	.495	.084	.084	.490	.092	.089	1.001	.089	.088
MI^*^/MI	−.086	.051	.061	.663	.100	.129	.372	.071	.072	.976	.079	.117
MI^*^/MI^*^	−.087	.051	.060	.674	.100	.126	.337	.077	.081	.970	.080	.112
IPW/MI^*^	−.003	.078	.076	.504	.086	.091	.427	.096	.089	.978	.092	.095
IPWe/MI	.003	.061	.060	.497	.081	.083	.491	.089	.087	1.001	.088	.089

MI*/MI gives biased estimation, because the imputation model at stage 1 is misspecified. Misspecification also of the imputation model at stage 2 (MI*/MI*) adds to the bias, especially in θ_4_. Bias also occurs when IPW is used at stage 1 instead of MI (IPW/MI*), but is smaller than that of MI*/MI*, and indeed of MI*/MI.

Theorems 1 and 2 of Robins and Wang (2000) enable the asymptotic (*N*→∞) percentage bias in 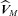 to be calculated when *M*=∞ (see Web Appendix). The asymptotic percentage bias in 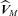 was 3.7% for θ_4_ and less than 1% for θ_0_, θ_2_, θ_3_, and θ_23_, which is in line with the finding above that 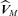 was approximately unbiased for finite *N* and *M*.

The results above were obtained using the true weights. In practice, weights would usually be estimated. Row IPWe/MI in Table 2 shows the results when weights are estimated. The variance estimators are approximately unbiased. Note that for IPWe/MI, 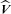 was replaced by a sandwich estimator that accounts for uncertainty in the weights (Robins et al., 1994). When 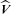 was instead used, the variance for θ_0_ was overestimated.

## 6. Application

The NCDS consists of 17,638 individuals born in Britain during one week in 1958 (Power and Elliott, 2006). 920 immigrants added later are not considered here. Data were collected at birth and at ages 7, 11, 16, 23, 33, and 45. A total of 16,334 nonimmigrants were still alive and free from type 1 diabetes at age 45 and of these, 8953 (55%) participated in a biomedical survey.

Thomas, Hypponen, and Power (2007) investigated the effect of characteristics measured at birth and adult adiposity (body mass index [BMI] and waist size at 45) on glucose metabolism at age 45. Subjects were classified as having high blood glucose if their glycosylated hemoglobin (A1C) was greater than 6% or they had type 2 diabetes. Immigrants and individuals with type 1 diabetes were excluded. Data on blood glucose, BMI and waist size at 45 were available for 7518 of the 8953 participants. Of these, 1845 (25%) had incomplete data on the factors measured at birth. Thomas et al., using the ice command in STATA (Royston, 2005), performed MI by chained equations (Van Buuren, 2007) on the 7518 subjects, producing 10 complete datasets. These 7518 were then analyzed as though representative of all 16,334 nonimmigrants alive and free from type 1 diabetes at age 45. Thomas et al. concluded that the factors measured at birth were related to blood glucose at 45 and that, moreover, some of these effects were largely mediated through adult adiposity.

We repeated this analysis but used IPW to allow the relation between glucose and the predictors to differ in the 7518 subjects with complete age 45 data from the other 8816 cohort members. Here stage 1 missingness refers to the age 45 data and stage 2 refers to the data measured at or before birth. Thomas et al. used a CC/MI analysis (i.e., used complete cases at stage 1 and MI at stage 2), whereas we use IPW/MI.

In the missingness model for stage 1, i.e., for the probability that at least one of glucose, BMI and waist size is missing, we used the potential predictors of missingness recorded at birth or age 7 identified by Atherton et al. (2008) and listed in their Table 3. We also used gestational age (< 38 versus ≥ 38 weeks) and a set of variables recorded at age 11: math and reading scores (normal/low), internalizing and externalizing problems (normal/intermediate/problem), and verbal and nonverbal scores (normal/low). All predictors were categorical, and most binary.

**Table 3:** *LOR and SEs for predictors of high blood glucose. Binary predictors are gestational age* < 38 *weeks, preeclampsia, smoking during pregnancy, prepregnancy BMI*≥ 25*Kg*/*m*^2^, *and manual socioeconomic position (SEP) at birth. Ordinal and continuous predictors are birth weight for gestational age (tertile), BMI at age 45 (Kg/m*^2^*), and waist circumference at age 45 (cm). Adjustment was also made for sex and family history of diabetes.*

	CC/MI		IPW/MI		MI/MI	
			
	LOR	SE	LOR	SE	LOR	SE
Short gestation	0.46	0.22	0.48	0.23	0.44	0.20
Preeclampsia	0.46	0.27	0.55	0.27	0.47	0.25
Mother overweight	0.29	0.15	0.36	0.16	0.18	0.12
Smoke in pregnancy	0.02	0.14	0.04	0.14	0.04	0.14
Manual SEP	0.37	0.17	0.44	0.18	0.39	0.17
Birth weight	−0.31	0.09	−0.31	0.09	−0.32	0.09
BMI age 45	0.04	0.02	0.02	0.02	0.03	0.02
Waist size age 45	0.07	0.01	0.07	0.01	0.07	0.01

Not everyone attended the age 7 and age 11 visits, and even those who did had some missing values. Therefore, some predictors of missingness at stage 1 were themselves missing. To deal with visit missingness, we partitioned the sample into four strata according to which of the age 7 and age 11 visits were attended. A different logistic regression was fitted to each stratum, using only predictors from the visits attended by individuals in that stratum. Missing values in these predictors were dealt with by introducing missing indicator variables. The missing indicator method can cause bias when used for variables in an analysis model (Jones, 1996). Although we are using it to calculate weights, not in the analysis model, this method is imperfect and we do not recommend it for general use. Therefore, we also calculated a second set of weights by multiply imputing missing predictors of missingness. The results obtained using this second set of weights were very similar to those (reported below) obtained using missing indicators.

The mean weight was 2.5; 5th and 95th percentiles were 1.6 and 5.2; the maximum was 23.1. As found by Atherton et al. (2008), disadvantaged individuals were more likely to be missing at stage 1. In the stratum who attend both age 7 and 11 visits, the following variables were significant at the 5% level: breastfed <1 month; mother leaving school at or before statutory age; short stature, overweight, internalizing, and externalizing problems at age 7; internalizing and externalizing problems, low math, low reading, and low nonverbal scores at age 11.

For stage 2 we used the same imputation model as Thomas et al., except that we included the weights. Following guidelines of White, Royston, and Wood (2010), 25 imputations were used. This MI model used only the variables in the analysis model and the weights. We also tried adding variables used as predictors in the missingness model to the imputation model, but this made very little difference to the results below.

Table 3 shows the estimated log odds ratios (LOR) and SEs. Due to the stochastic nature of MI and the inclusion of weights in the imputation model, the results for CC/MI are slightly different (maximum difference 0.03) from those reported by Thomas et al. (2007). As can be seen, using IPW at stage 1 (IPW/MI) does not substantially change the results. The biggest differences are that ORs for preeclampsia, mother overweight, and manual class have risen slightly, and the first two have changed from being almost significant to just significant. SEs are also slightly larger.

We investigated why these ORs increased slightly when weighting was used. The missingness model indicated that disadvantaged individuals were more likely to be missing at stage 1. Therefore, using IPW gives more weight to disadvantaged individuals. We partitioned the stratum who attended both age 7 and age 11 visits into two groups, advantaged and disadvantaged, using the following rule: individuals with at least three of the following indicators of disadvantage were classified as disadvantaged: breastfed < 1 month; mother leaving school early; short stature, overweight, internalizing, and externalizing problems at age 7; and internalizing and externalizing problems, and poor math, reading, and nonverbal scores at age 11. Using this rule, the disadvantaged group contained 29% of individuals. The other 71% were classified as advantaged. The analysis model was fitted to the two groups separately. The LORs for preeclampsia, mother overweight, and social class were 0.59, 0.70, and 0.33, respectively, in the disadvantaged group, and −0.04, −0.04, and 0.41 in the advantaged group. Therefore, the observed relation between glucose and preeclampsia/overweight is stronger in the disadvantaged individuals. It seems likely therefore that the reason why ORs for preeclampsia and overweight in the whole cohort are greater when IPW is used (IPW/MI versus CC/MI) is that IPW gives more weight to the disadvantaged group. The relation between manual class and glucose, however, is slightly weaker in the disadvantaged group, leaving its increased OR unexplained.

Assuming then that the probability that glucose, BMI and waist size at 45 years are complete does not depend on variables in the analysis model given available predictors of missingness, the associations found by Thomas et al. in the sample of 7518 individuals do generalize to the population of nonimmigrants still alive and free from type 1 diabetes at age 45.

Finally, we used MI/MI, i.e., imputed all missing values for all 16,334 individuals. Included in the imputation were the variables in the analysis model and the predictors in the missingness model of IPW/MI. A total of 100 imputed datasets were created. Table 3 shows the results. They do not differ substantially from those of IPW/MI. Some SEs are slightly smaller. The small increases in the ORs of preeclampsia and mother overweight seen in IPW/MI relative to CC/MI are not replicated. In fact, the OR of overweight is lower in MI/MI than in CC/MI.

To investigate why, we partitioned the 12,501 individuals who attended both age 7 and age 11 visits into four groups, using the same rule for disadvantage as before: disadvantaged with observed glucose; disadvantaged with imputed glucose; advantaged with observed glucose; and advantaged with imputed glucose. The analysis model was fitted to each group separately. It was found that, whereas the relation between blood glucose and its predictors differed considerably between the advantaged and disadvantaged groups in the set of individuals whose glucose was observed, this difference was not seen in those with imputed glucose. In particular, the LORs for overweight were 0.57 and −0.17 in the disadvantaged and advantaged groups with observed glucose, respectively, but were 0.15 and 0.18 for those with imputed glucose. Interaction terms are needed in the imputation model, e.g., imputation could be done separately in the two groups. Careful assessment of the imputation model might have revealed this, but such assessment might not always be made.

## 7. Discussion

Robins and Wang (2000) derive a general formula for the asymptotic variance of an MI estimator based on a complete-data estimator solving a set of estimating equations. This formula applies when improper imputation and a parametric imputation model are used. IPW/MI could be carried out in this way and the Robins and Wang (2000) variance formula used. The formula is, however, complicated and has not been implemented in standard software. Using proper imputation with Rubin’s rules is appealing because it is simpler and can be used with nonparametric imputation procedures. Robins and Wang (2000) also give a formula for the asymptotic bias of the Rubin’s rules variance estimator when *M*=∞. We used this to show that, in the case of linear regression with MI of a missing outcome, the Rubin’s rules variance estimator for IPW/MI is consistent when *M*=∞. We also used it in the setting described in Section 5, where a missing covariate is imputed. The expression derived for the asymptotic bias in the Rubin’s rules variance estimator for IPW/MI was complicated and did not reduce to zero. However, both the asymptotic and finite-sample biases were found to be small in this study. In the Web Appendix, we describe two simulation studies of logistic regression, one with an imputed outcome and one with an imputed covariate. In both, the Rubin’s rules variance estimator was approximately unbiased. Schafer (2003) comments that “although we may find it difficult to prove good performance for [MI using a nonmaximum likelihood estimator], that does not imply that good performance will not be seen in practice. Experience suggests that Bayesian MI does interact well with a variety of semi- and nonparametric estimation procedures.”

If the weights are just sampling weights, they will be known, but if they are used to account for missing data, they will need to be estimated. A limitation of our proof in Section 4 is that the complete-data variance estimator assumes that weights are known and ignores any estimation uncertainty about them. This uncertainty is commonly ignored, thus overestimating the variance (Robins et al., 1994), as we saw in Section 5. If software allows, we recommend using a sandwich estimator that accounts for the uncertainty in the weights (Robins et al., 1994).

Some researchers may prefer to use straightforward MI (what we called MI/MI). Provided that the imputation models are correctly specified, this will be more efficient than IPW/MI. However, our (admittedly contrived) simulations and (not contrived) real data example have shown that those who prefer IPW/MI have some justification for their caution. A possible use for IPW/MI is as a check, or diagnostic, for MI/MI. If the results of IPW/MI and MI/MI are very different, further exploration would be warranted, possibly leading to refinement of the imputation model. We have not considered the effect of misspecified missingness models. Such misspecification would typically cause bias, just like misspecification of the imputation model in MI/MI. However, the fit of the missingness model, which is a model for a univariate response, is easier to assess, and more able to be assessed (Vansteelandt, Carpenter, and Kenward, 2010), than that of a complex multivariate imputation model. Furthermore, IPW/MI is needed when sampling weights are used, even if all missing values are imputed.

IPW/MI will be most appealing when the model for the weights is relatively simple compared with the imputation model. This will not always be so. Also, a limitation of all IPW methods is their difficulty in handling nonmonotone missingness in the predictors in the missingness model. Robins and Gill (1997) propose a procedure for handling such missingness, but this is complicated to use and limited in practice to a small number of missing predictors.

Another alternative to IPW/MI is IPW/IPW. This is simpler, but has the disadvantage that an individual is excluded from an analysis even if he/she is missing just one variable. Furthermore, if multiple analyses are being performed with different variables, either a different set of weights is needed for each analysis (because an individual who is complete for one analysis may be incomplete for another) or a single set of weights is calculated but only for individuals who are complete cases for all the analyses (Goldstein, 2009). IPW/MI, on the other hand, would allow a single set of weights to be used, as imputation could ensure that the set of complete cases were the same for each analysis.

## 8. Supplementary Materials

The Web Appendix referenced in Sections 2, 3, 5, and 7 is available under the Paper Information link at the *Biometrics* website http://www.biometrics.tibs.org.
